# Could nutritional supplements act as therapeutic adjuvants in COVID-19?

**DOI:** 10.1186/s13052-021-00990-0

**Published:** 2021-02-15

**Authors:** Giorgio Costagliola, Erika Spada, Pasquale Comberiati, Diego G. Peroni

**Affiliations:** Clinical and Experimental Medicine, Division of Pediatrics, University of Pisa, Via Roma 57, 56126 Pisa, PI Italy

**Keywords:** COVID-19, Cytokine storm, Immune system, Lactoferrin, Nutrition, Probiotics, Supplementation, Vitamin d, Zinc

## Abstract

**Background:**

The role of the immune system and inflammatory response in the pathogenesis of the severe manifestations of coronavirus disease 2019 (COVID-19) is well known. Currently, different therapies active on the immune system are used for the management of COVID-19. The involvement of the immune system also opens the opportunity for the use of nutritional supplements with antimicrobial and immunomodulatory activity.

**Main aspects:**

Nutritional supplements with antimicrobial and immunomodulatory activity are promising therapeutic adjuvants for the treatment of COVID-19, and also for the prevention of viral spreading. In particular, the role of vitamin D, probiotics, lactoferrin, and zinc is of significant clinical interest, although there are only a few data on their use in COVID-19 patients. Their molecular actions, together with the results of studies performed on other respiratory infections, strongly suggest their potential utility in COVID-19. This article discusses the main properties of these nutritional supplements and their potential applicability in the prevention and treatment of COVID-19.

**Conclusion:**

The supplementation with vitamin D, probiotics, lactoferrin and zinc could have a role both in preventing SARS-CoV-2 infection and in mitigating the clinical course in infected patients, contributing in the prevention of immune-mediated organ damage.

## Background

The immune system and the inflammatory response play an important role in determining the disease course of coronavirus disease 2019 (COVID-19). An adequate innate and adaptive immune response is fundamental to allow the clearance of the infectious agent, but an overactive immune and inflammatory response can be responsible for some of the disease manifestations. In particular, in patients with severe manifestations of COVID-19, as vasculitis, acute respiratory distress syndrome (ARDS), and septic shock, high levels of pro-inflammatory cytokines are evidenced, in a condition defined as “cytokine storm”, central in the pathogenic mechanism of such manifestations [1]. The immune dysregulation has a peculiar clinical relevance in children, which can develop a potentially life-threatening condition defined as “multisystem inflammatory syndrome in children” (MIS-C) after SARS-CoV-2 infection [2]. In both adult and pediatric patients, the current approaches to control the inflammatory response are represented by drugs used in other immune-mediated diseases (corticosteroids, intravenous immunoglobulin, anakinra, canakinumab, tocilizumab) [1]. Given the importance of a “balanced” immune and inflammatory response, able to control the infection avoiding the risk related to the uncontrolled inflammation, the potential utility of different nutritional supplements with antiviral, immunostimulating and immunomoldulatory properties has been suggested by different authors [3]. In this paper, we discuss the promising role of supplementation with vitamin D, probiotics, lactoferrin, and zinc as adjuvants in the management of COVID-19.

### Vitamin D

Vitamin D (VD) has well-known antimicrobial and immunomodulatory activities, and it is used as an adjunctive treatment to reduce the incidence and severity of different conditions, including influenza and recurrent respiratory infections (RRI) [4]. VD supplementation has demonstrated efficacy in the prevention of acute respiratory infections, with the most significant results observed in patients receiving daily or weekly VD doses [5]. Moreover, different authors have demonstrated that people with VD insufficiency (25OHD < 30 ng/ml) or deficiency (25OHD < 20 ng/ml) have a higher risk of developing respiratory infections [6]. With specific regard to COVID-19, it is important to underline that the disease has mainly spread in winter when is more probable to observe VD deficiency and that the severity of COVID-19 has shown a north-south gradient, being more pronounced in latitudes associated with a deficient VD status^4^. Also, the severity of COVID-19 can be higher in patients with VD deficiency, as suggested by a retrospective analysis performed on patients with respiratory failure [7]. The antimicrobial action of VD depends mainly on the activation of the innate and adaptive immune response, facilitating the elimination of the infectious agent. From a molecular point of view, VD promotes the differentiation of monocytes into macrophages, enhances chemotaxis, leukocyte recruitment, and the antimicrobial activity of cells of the innate immune system. Moreover, it promotes the production and release of defensins and cathelicidin and reinforces the barrier function of different organs [8]. This last point is of extreme interest, as could partly explain the role of VD in the reduction of the transmission of infectious diseases, including COVID-19. The immunomodulatory role of VD is complex, as the VD receptor is expressed on a wide variety of immune cells, influencing the secretion of cytokines and the function of different lymphocyte subpopulations [9]. In particular, VD reduces the production of immunoglobulin, the T-helper 1 (Th1) and T-helper 17 (Th17) response, decreasing the release of pro-inflammatory cytokines, and promotes the proliferation of regulatory T cells (Tregs) and the development of a T-helper 2 (Th2) response [9–11]. The effects of VD on lymphocytes are partly mediated from the influence on the function of antigen-presenting cells [9], with the induction of a “tolerogenic” phenotype in dendritic cells (DCs) [11]. Interestingly, the influence of VD on DCs and T cell proliferation has been demonstrated also in patients with primary immunodeficiencies affecting the T cell response [12], which represent a population carrying an increased risk of developing immune dysregulation and severe COVID-19. Therefore, its wide-spectrum immunomodulatory action allows the hypothesis of the utility of VD supplementation in COVID-19, both for the prevention of the progression to the severe manifestations of the disease and for the reduction of the transmission of the infectious agent. Data deriving from previous studies analyzing the role of VD supplementation in the prevention and treatment of respiratory infections are markedly heterogeneous, as the baseline VD levels of the population examined and the posology of VD are not standardized [6]. However, in most of the trials on pediatric patients, the intervention consists of a daily intake of 400–1200 IU of VD [5, 6, 13]. Therefore, a supplementation within this posology range, individualized considering the patient’s risk factor and baseline 25OHD levels, can be suggested for the prevention of COVID-19 (Table 1). On the other hand, the use of short-term regimens of high-dose VD in patients with respiratory infections has not demonstrated a significant beneficial effect [6]. The importance of VD supplementation could be even more pronounced during winter and in geographic areas at a high prevalence of VD deficiency.

**Table 1:** Suggested posology for the use of nutritional supplements in the prevention of COVID-19

Nutritional supplement	Suggested posology
Vitamin D	400–1200 IU daily for 6–12 months In patients with VD deficiency or insufficiency, consider higher doses
Lactoferrin	100–500 mg daily for 3–6 months Consider doses up to 600 mg daily in adults and adolescents
Zinc	10–20 mg daily for 6–12 months Consider the use of 30 mg daily during autumn and winter

In absence of definitive data on the prevention of COVID-19, the posology ranges suggested in this table derive from the experience in the prevention of other viral infections

### Probiotics

The role of gastrointestinal and lung microbiome and the potential utility of supplementation with probiotics in COVID-19 are not completely defined, although results from studies on other infectious diseases seem to suggest a beneficial effect. Probiotics have shown efficacy in the prevention of RRI, infectious gastroenteritis and sepsis, and have also been associated with a reduced incidence of ventilator-associated pneumonia in patients hospitalized in intensive care units [14–16]. In COVID-19, probiotics could have a therapeutic effect through different molecular mechanisms, influencing both the transmission of SARS-CoV-2 and the immune balance of the host. As the infectious agent is transmitted also through feces, particularly in children, the administration of probiotics could interfere with this mechanism reinforcing the gut epithelial barrier and directly competing with the proliferation of SARS-CoV-2 [15]. Moreover, specific probiotics are also able to enhance local and systemic immune response, involving also the respiratory system through a complex network of interactions with the immune system and the respiratory microbiome, creating a “gut-lung axis” which finally favors the clearance of the infectious agent [17, 18]. Indeed, influencing the gut microbiome can lead to increased local and systemic production of different pro-inflammatory cytokines with antiviral activity, like type 1 interferons. Moreover, probiotics can enhance the activity of innate and adaptive immune system with different molecular mechanisms, including the increased function of toll-like receptors (TLR) and the influence on the function of antigen-presenting cells [17]. Apart from the molecular mechanisms, which suggest a potential utility of probiotic supplementation in COVID-19, interesting data derive also from patients with influenza infection. In these patients, the administration of probiotics is associated with clinical improvement, with the increased humoral and cellular adaptive immune response against the virus, and also with a better humoral immune response after vaccination [15, 19]. Finally, the microbiome has a role in the modulation of immune and inflammatory responses. It influences the balance between Th17 cells (with pro-inflammatory activity) and Tregs (anti-inflammatory), thus preventing overactivation of the immune system [17, 18, 20]. Therefore, in COVID-19 probiotics could reduce the systemic levels of pro-inflammatory cytokines, associated with the “cytokine storm”, and cause an elevation of serum anti-inflammatory cytokines, such as IL-10 [15]. Although it represents a promising adjuvant therapy, further studies are needed to define the population which could benefit from the administration of probiotics. Also, the strain of probiotics (or a mixture of strain) to be used, the posology and the duration of the intervention have to be better defined. In this regard, it is important to underline that most of the studies on the prevention of pediatric respiratory infections are performed using *Lactobacillus* strains, *Bifidobacterium* strains, or mixtures of strains, for a variable duration, which is usually between 3 and 12 months [21].

### Lactoferrin

Lactoferrin is a glycoprotein physiologically present in mammalian milk, with a higher concentration in colostrum. It represents a central component of the innate immune response in the newborn, having antimicrobial and anti-inflammatory properties. For these effects, lactoferrin is currently administered as a nutritional supplement in preterm newborns to prevent the development of neonatal sepsis, with demonstrated beneficial effects [22]. Moreover, supplementation with lactoferrin is associated with a preventive effect against the development of different viral infections [23]. For what concerns COVID-19, an important point sustaining the potential utility of lactoferrin is the broad-spectrum antiviral activity, demonstrated in studies on other viral respiratory infections, including SARS-CoV, influenza A virus, parainfluenza virus, adenovirus and respiratory syncytial virus (RSV) [24, 25]. The antiviral effect depends on multiple molecular mechanisms, including the impairment of the viral anchoring on the cellular surface, by blocking the interaction between the virus and heparin sulfate glycosaminoglycan, and the inhibition of the viral replication [24–26]. The latter effect is enhanced in presence of high concentrations of zinc, thus suggesting the utility of combined supplementation of zinc and lactoferrin in COVID-19 [23]. Lactoferrin facilitates the clearance of the infectious agent also stimulating the innate immune response, and particularly enhancing the activity of macrophages, neutrophils and natural killer cells [26]. The action of lactoferrin on DCs facilitates the antigen presentation to T cells, thus contributing to the defense against viral and bacterial infections [27, 28].

Beyond the antimicrobial activity, lactoferrin carries a significant anti-inflammatory and immunomodulatory effect. The molecular mechanism involves the modulation of the secretion of different pro-inflammatory cytokines, including IL-6, which has a pivotal role in the pathogenesis of ARDS and MIS-C in pediatric patients with COVID-19 [29]. Moreover, lactoferrin can decrease the expression of different chemotactic factors and adhesion molecules (ICAM-1, E-selectin) and reduce the damage deriving from the production of reactive oxygen species [28–30]. Therefore, lactoferrin acts on the viral adhesion and replication, important in controlling the first phase of the infectious process, and also modulating the inflammatory response in infected patients. These data support its potential utility in the prevention of the infection by SARS-CoV-2 and, in infected patients, in the mitigation of the clinical course, avoiding the development of ARDS. Studies on the prevention and treatment of viral infections in children using lactoferrin are limited. The administration of 100–500 mg daily of lactoferrin in children over 3–6 months showed promising results in the reduction of frequency and severity of viral gastroenteritis, and a posology of 600 mg daily reduced the incidence of cold in adult patients [23]. As a consequence, the use of similar posologies could be a beneficial approach during the COVID-19 pandemic (Table 1).

Finally, lactoferrin could represent one of the factors contributing to the lower incidence and severity of COVID-19 in infant age, and specifically the reduced incidence of clinically relevant disease in the newborn. For the high values of lactoferrin in colostrum, early breastfeeding could represent a useful measure to prevent COVID-19 in newborns [26, 27, 29].

### Zinc

Zinc is an element with a central role in several cellular processes, including multiple signaling pathways involved in the activity of innate and adaptive immune response, and is pivotal in allowing the therapeutic action of different drugs, including pharmacologic agents used in the treatment of COVID-19. Moreover, it is an essential component of a wide range of enzymes and can directly influence the function and permeability of the cell membrane [31]. Therefore, a potential role for the supplementation of zinc in patients with COVID-19 has been proposed, with a particular focus on populations with a higher risk of zinc deficiency, defined as zinc plasmatic levels below 60 mcg/dL [6]. Zinc deficiency is more frequently observed in elderly patients, or individuals affected by chronic cardiac or pulmonary disease, hypertension and diabetes, while this finding in pediatric age is less common [32]. Interestingly, in elderly patients, the supplementation with zinc has demonstrated to be safe and effective in the reduction of the total number of infections [32]. As in children and adolescents the incidence of zinc deficiency is low, is reasonable to hypothesize that adequate concentrations of zinc can contribute to determining the reduced incidence and severity of COVID-19 in this age subgroup. Zinc has a direct antiviral activity, as studies in vitro demonstrated inhibition of viral replication on different pathogens, including SARS-CoV and RSV, through the interference with the function of RNA-dependent RNA polymerase [32, 33]. This molecular mechanism suggests that high intracellular concentrations of zinc could have a positive effect on the clearance of SARS-CoV-2, with a beneficial clinical impact. Zinc participates in the control of the infection also stimulating the activation of the antiviral immune response, through the enhancement of the production of pro-inflammatory cytokines, particularly interferon, and acute-phase reagents and the promotion of the proliferation of cells involved in the innate and adaptive immune response [33]. Concerning adaptive immunity, zinc is an essential co-factor for the enzyme thymulin, which promotes the proliferation and differentiation of T cells, while evidence of a direct influence on B cell functioning is lower [31, 34].

Another interesting point is that zinc could have a role in controlling and reducing organ damage secondary to the inflammatory response to SARS-CoV-2. Indeed, it activates the transcription factor FOXP3, implicated in the differentiation of Tregs (which produce anti-inflammatory cytokines such as IL-10), regulates the Th1/Th2 balance, the proliferation of Th17 cells and participates in the minimization of the organ damage caused by reactive oxygen species [31, 34].

Finally, zinc is an important adjuvant of different therapeutic agents used in COVID-19. It has a particular efficacy demonstrated, in vitro, in the amplification of the antiviral activity, with increased apoptosis of infected cells, of chloroquine and hydroxychloroquine [35], suggesting that patients being treated with antimalarial drugs could benefit from zinc supplementation.

The results from studies on the use of zinc in both prevention and treatment of respiratory infections are not univocal. However, as different authors reported a reduction of the infectious rate after the supplementation with a daily dose of 10–20 mg of zinc for 6–12 months [6], it is reasonable to suggest that this intervention can help also in the prevention of COVID-19 (Table 1). Although evidence is lower [6], the administration of zinc as a therapeutic adjuvant in symptomatic COVID-19 patients could be considered to reduce the duration of the disease.

### Concluding remarks

From the analysis of the current evidence, it is reasonable to conclude that supplementing vitamin D, probiotics, lactoferrin and zinc may have a rationale as an adjuvant treatment in COVID-19, and also in the prevention of viral spreading. Although data from experimental studies are lacking and further studies are expected, their molecular mechanisms of action, together with the efficacy demonstrated in other viral infections, including SARS, represent the rationale to support their administration in COVID-19 prevention.

## Data Availability

Not applicable.

## Abbreviations

ARDS: acute respiratory distress syndrome
COVID-19: coronavirus disease 2019
DCs: dendritic cells
MIS-C: multisystem inflammatory syndrome in children
RRI: recurrent respiratory infections
RSV: respiratory syncytial virus
Th: T-helper
Tregs: regulatory T cells
VD: Vitamin D
